# Flattening filter‐free accelerators: a report from the AAPM Therapy Emerging Technology Assessment Work Group

**DOI:** 10.1120/jacmp.v16i3.5219

**Published:** 2015-05-08

**Authors:** Ying Xiao, Stephen F. Kry, Richard Popple, Ellen Yorke, Niko Papanikolaou, Sotirios Stathakis, Ping Xia, Saiful Huq, John Bayouth, James Galvin, Fang‐Fang Yin

**Affiliations:** ^1^ Department of Medical Physics Thomas Jefferson University Hospital Philadelphia PA; ^2^ Department of Radiation Physics University of Texas MD Anderson Cancer Center Houston TX; ^3^ Department of Radiation Oncology University of Alabama Birmingham Birmingham AL; ^4^ Department of Medical Physics Memorial Sloan‐Kettering Cancer Center New York NY; ^5^ Department of Medical Physics University of Texas Health Science Center at San Antonio San Antonio TX; ^6^ Department of Radiation Onoclogy University of Texas Health Science Center at San Antonio San Antonio TX; ^7^ Department of Radiation Oncology The Cleveland Clinic Cleveland OH; ^8^ Department of Radiation Oncology University of Pittsburgh Medical Center Pittsburgh PA; ^9^ Department of Human Onoclogy University of Wisconsin Madison WI; ^10^ Department of Radiation Oncology Jefferson Hospital Newtown PA; ^11^ Department of Radiation Oncology Duke University Medical Center Durham NC USA

**Keywords:** flattening filter free, AAPM, FFF, calibration, commissioning, safety, QA

## Abstract

This report describes the current state of flattening filter‐free (FFF) radiotherapy beams implemented on conventional linear accelerators, and is aimed primarily at practicing medical physicists. The Therapy Emerging Technology Assessment Work Group of the American Association of Physicists in Medicine (AAPM) formed a writing group to assess FFF technology. The published literature on FFF technology was reviewed, along with technical specifications provided by vendors. Based on this information, supplemented by the clinical experience of the group members, consensus guidelines and recommendations for implementation of FFF technology were developed. Areas in need of further investigation were identified. Removing the flattening filter increases beam intensity, especially near the central axis. Increased intensity reduces treatment time, especially for high‐dose stereotactic radiotherapy/radiosurgery (SRT/SRS). Furthermore, removing the flattening filter reduces out‐of‐field dose and improves beam modeling accuracy. FFF beams are advantageous for small field (e.g., SRS) treatments and are appropriate for intensity‐modulated radiotherapy (IMRT). For conventional 3D radiotherapy of large targets, FFF beams may be disadvantageous compared to flattened beams because of the heterogeneity of FFF beam across the target (unless modulation is employed). For any application, the nonflat beam characteristics and substantially higher dose rates require consideration during the commissioning and quality assurance processes relative to flattened beams, and the appropriate clinical use of the technology needs to be identified. Consideration also needs to be given to these unique characteristics when undertaking facility planning. Several areas still warrant further research and development. Recommendations pertinent to FFF technology, including acceptance testing, commissioning, quality assurance, radiation safety, and facility planning, are presented. Examples of clinical applications are provided. Several of the areas in which future research and development are needed are also indicated.

PACS number: 87.53.‐j, 87.53.Bn, 87.53.Ly, 87.55.‐x, 87.55.N‐, 87.56.bc

## I. INTRODUCTION

Photon beams are generated by bombarding a high‐Z target with a high‐energy electron beam. The resultant megavoltage bremsstrahlung beams present a bell‐shape profile with the highest intensity at the center. In conventional linear accelerators, uniform intensity across the treatment field (at least nominally) is obtained by placement of a flattening filter in the beam. However, modern radiotherapy practice now routinely utilizes fluence modifying techniques, such as intensity‐modulated radiation therapy (IMRT), to create more conformal dose distributions. In such cases, the flattening filter becomes unnecessary in the beam production process. Additionally, for small fields (such as are used in stereotactic procedures), even without the flattening filter the treatment field is nearly flat over the central few centimeters, again rendering the flattening filter unnecessary; furthermore, as neither patients nor targets are flat, flattening filter‐free (FFF) fields may be useful for moderate or even large targets.

Clinical use of FFF beams was initially driven by the attempt to reduce the long delivery time required for SRS treatments, as removing the flattening filter increases the dose rate by a factor of 2–4. The increased intensity associated with FFF beams is particularly useful for small field stereotactic radiosurgery (SRS) and/or stereotactic body radiation therapy (SBRT) procedures,^(1)^ but may be useful for a wide range of fields and treatments.^2^, ^3^ Other early clinical applications were also reported using FFF technology to improve the delivery efficiency,^4^, ^5^, ^6^ evolving into studies of stereotactic body radiation therapy (SBRT),^7^, ^8^, ^9^, ^10^ all of which demonstrated positive results. FFF technology has been in clinical use for many years, starting with the Scanditronix racetrack microtron MM50.^11^, ^12^ More modern linear accelerators that omit the flattening filter include the helical TomoTherapy machine, as well as CyberKnife system.^13^, ^14^, ^15^ It is, therefore, no surprise that mainstream linear accelerators now include this as standard product offering.

FFF beams have many distinct characteristics compared to conventional photon beams. They have a different beam profile and higher dose rate, but also a different photon energy spectrum and different head‐scatter properties. Consequently, there are unique features to FFF beams, including properties of the beams (sharper penumbra, less head scatter, and less out‐of‐field dose), dosimeter response (increased ion recombination), vault shielding, and possibly even radiobiology. At present, hundreds of medical linear accelerators with FFF functionality have been installed. However, general guidelines regarding the acceptance, commissioning, and quality assurance have not been compiled. This document provides a technical overview of current FFF technology, and offers guidelines and recommendations for dose‐calculation, acceptance testing, commissioning, quality assurance, facility planning, radiation safety, and clinical implementation.

## II. TECHNICAL ISSUES

### A. Machine Overview

There are multiple commercial implementations of FFF technology now available and a review of the treatment head physics including monitor chamber and steering effects, X‐ray spectra, photon and electron characteristics, and neutron production has recently been published.^16^, ^17^ The following sections supplement this work by providing technical summaries of commercial systems available at the time of this report.

#### A.1 Varian

Varian Medical Systems offers 6 and 10 MV FFF beams on the TrueBeam linear accelerator, in addition to the conventional flattened beams. Varian refers to the FFF beams as “High Intensity Mode.” Beam characteristics are presented in Table 1. In their implementation, the same electron beam is used to create both flattened and FFF beams of the same nominal energy. Therefore, electron energy at the target is the same and removal of the flattening filter increases output, but also reduces the penetrative quality of the photon beam due to the reduced beam hardening.

**Table 1:** Characteristics of commercially available FFF beams. All dosimetric quantities are given for a $10\times 10\;{\text{cm}}^{2}$ field at 100 cm SSD unless otherwise noted and were provided by the manufacturers.

	*Varian*		*Elekta*		*Siemens*			
Nominal energy (MV)	6 FFF	10 FFF	6 FFF	10 FFF	7 UF	11 UF	14 UF	17 UF
Bremsstrahlung target material	Tungsten		Tungsten		Tungsten			
Approximate mean electron energy on target (MeV)	6.2	10.5	7	10.5	8.9	14.4	16.4	18.3
Filtration	0.8 mm Brass		2mm Stainless steel		1.27 mm Al			
$d_{\text{max}}$ (cm)	1.5	2.3	1.7	2.4	1.9	2.7	3.0	3.3
Dose at 10 cm depth (%)	64.2	71.7	67.5	73.0	68.5	74.5	76.5	78.0
Dose 10 cm from central axis ($40\times 40\;{\text{cm}}^{2}$ field), at $d_{\text{max}}$ (%)	77	60	70^a^	59^a^	68	57	–	–
Maximum dose rate on beam axis at $d_{\text{max}}$ (cGy/min)	1400	2400	1400	2200	2000	2000	2000	2000
Dose per pulse on beam axis at $d_{\text{max}}$ (cGy/pulse)	0.08	0.13	0.06	0.09/0.14^b^	0.13	0.13	0.13	0.13

^a^ Defined at 90 cm SSD, 10 cm depth

^b^ Feedback/nonfeedback machine.

Both the 6 and 10 MV FFF beams use a tungsten target. On FFF machines, the carousel that holds the flattening filters also has a position containing a 0.8 mm thick brass plate that is used for the FFF beams in place of a flattening filter to filter electrons and low‐energy photons.^(18)^

The monitor chamber is comprised of two separately sealed, positive‐pressure chambers. Whereas the traditional C‐series linac had four sectors per chamber, the TrueBeam accelerator includes a fifth, central, sector that evaluates coarse flatness. The primary chamber monitors dose rate and radial profile shape. The secondary chamber monitors dose rate and transverse profile shape. The beam monitoring system digitizes and counts every beam pulse and generates corrective feedback to beam steering servo systems to control the profile shape. For FFF beams, symmetry is actively controlled by the beam steering systems.

#### A.2 Elekta

Elekta offers 6 and 10 MV FFF beams on the Versa HD linear accelerator, in addition to the conventional flattened beams. Elekta refers to the FFF beams as “High Dose Rate Mode” (characteristics presented in Table 1).

In contrast to the Varian linear accelerator, the same electron beam is *not* used to create the FFF and corresponding flattened beam. Each of the beams on the Elekta family of linacs is defined by its own independent set of parameter values, referred to as its ‘Energy Set’. Each Energy Set includes the radio frequency and gun settings that define the electron beam energy and also the dosimetry calibration settings. Each of the FFF energies has its own independent Energy Set different from any flattened beam. This allows the penetrative quality of the FFF beams to be restored to the nominal value for that energy.

In the linac head, the bremsstrahlung beam is filtered with a stainless steel disc instead of the usual flattening filter. In addition to removing low‐energy photons, this also produces electrons that provide signal to the ion chambers in the treatment head that measure dose and other beam parameters. Two ion chambers provide independent measurements of dose, and a third contains a set of six collection plates arranged geometrically to provide uniformity signals for use by the beam control servo system that ensures beam stability.

#### A.3 Siemens

Although no longer in the radiotherapy market, many Siemens FFF machines are in clinical use, and so their properties are discussed here. Siemens offers a high‐intensity unflat (UF) beam in four photon energies: 7 UF, 11 UF, 14 UF, and 17 UF, in addition to the conventional flattened beams. At the time of this report, Siemens is the only manufacturer that offers a high‐energy (>10 MV) FFF beam. Available properties of the Siemens FFF beams are presented in Table 1.

Similar to the Elekta linac, the electron energy at the target for the FFF beams is different from the corresponding flattened beam; the 7 UF beam has a different accelerating potential than the flattened 6 MV beam (selected to result in a similar depth‐dose curve). Nomenclature for the FFF energies was also selected in an effort to minimize the accidental use of an FFF beam in place of a flattened beam. For example, 11 MV UF would be distinct from 10 MV flattened, although the two beams may have very similar radiological characteristics on the central beam axis.

Two sealed dose chambers, named Monitor_1 and Monitor_2 perform photon dosimetry in Siemens linacs. The function of dose Monitor_1 is to measure each dose pulse. Monitor_2 consists of five chambers that are used for measuring each dose pulse (redundant to Monitor_1) and monitoring off‐axis characteristics.^(17)^

### B. Acceptance testing

Acceptance testing for a linear accelerator without a flattening filter is similar to that of a conventional linear accelerator. The guidelines described in AAPM TG‐45^(19)^ and TG‐142^(20)^ reports should be followed, with the sole exception relating to the testing of those properties related to the beam flatness. Instead of measuring the flatness, the shape of the beam profile must be verified to ensure that it meets the machine performance specifications provided by the manufacturer. The details of these specifications, such as the number and location of profile points to be checked, are dependent on the agreement between the hospital and manufacturer at the time of purchase; however, specifications for beam profile shape should not vary substantially between flattened and FFF beams. Beam shape and symmetry measurements will typically be obtained using either a scanning system in water or film.^(19)^ Because of the higher dose per pulse of FFF beams, dose rate dependent measurement devices should be evaluated for possible effects, as described in C.1, C.2.

### C. Commissioning

#### C.1 Calibration

A standard calibration protocol, such as TG‐51^(21)^ or TRS‐398,^(22)^ should be followed. With the removal of the flattening filter, four special considerations, detailed below, exist due to differences in the beam quality, dose per pulse, and profile shape. These issues are also addressed in the AAPM Working Group on TG‐51 report.^(23)^

First, for a Varian implementation of the FFF beams, the softer photon spectrum results in a different beam quality conversion factor, $k_{Q}$, than was used for flattened beams. More generally, because of the different photon spectrum for any FFF beam, the beam‐quality specifier for a FFF beam, as compared with a flattened beam, has a different relationship with Spencer‐Attix stopping‐power ratios.^(24)^ However, the standard relationship between %*dd*(10)x and the ratio of $(L/{\rho )}_{\text{air}}$ to $(L/{\rho )}_{\text{water}}$, which is used in the TG–51 protocol to calculate the quality conversion factor $k_{Q}$, is acceptable for beams with or without a flattening filter with a maximum error of 0.4%. That is, $k_{Q}$ may be slightly different for FFF beams, but can be determined in the same manner as was done for flattened beams. It is also important to note that the validity of this method to determine $k_{Q}$ only applies to chambers with low‐Z or aluminum electrodes; chambers with high‐Z electrodes should not be used for reference dosimetry in FFF beams.^23^, ^25^ As an alternative approach to specifying beam quality, studies have also evaluated using TPR10/20.^(26)^ It was found that, if TPR10/20 is used, there are two different relationships between the ratio of $(L/{\rho )}_{\text{air}}$ to $(L/{\rho )}_{\text{water}}$ which differ by 0.4%–1%. When using TPR10/20 in a beam without a flattening filter, it is necessary to subtract 0.5% from the value of $k_{Q}$ for a given value of TPR10/20.^(27)^ Due to this complication, this method should be used with caution.

Second, it is recommended in TG‐51^(21)^ that, when determining $k_{Q}$ at high energies (about 10 MV and above), 1 mm of lead be used to reduce electron contamination at $d_{\text{max}}$ to a negligible level. It is unclear if FFF beams at 10 MV require the use of lead. The prudent and appropriate approach is to use lead unless it has been verified to be unnecessary for a given beam (that is, the same value of $k_{Q}$ is produced).

Third, the elevated dose per pulse of FFF beams results in increased ion recombination and therefore a larger correction factor, $P_{ion}$.^28^, ^29^ However, it is likely that $P_{ion}$ can be calculated using the same two‐voltage formalism that is presented in TG‐51 for flattened beams — this approach was found to be accurate within 0.3% for common Farmer‐type chambers, and offers comparable accuracy to that seen for flattened beams.^(28)^ However, per the AAPM Working Group on TG‐51 report, the suitability of the two‐voltage technique should be verified for all reference dosimetry scenarios, including FFF (and flattened) beams.^(23)^ This can be done through the use of Jaffe plots (1/current (or charge) vs. 1/voltage) to determine the relationship between the ionization chamber bias and signal lost to recombination and, therefore, the validity of the two‐voltage $P_{\text{ion}}$. The correction factors of several commonly used ionization chambers for Varian 6 MV and 10 MV FFF beams are shown in Table 2. Of note, $P_{\text{ion}}$ is determined primarily by the instantaneous dose rate (the dose per pulse), which usually doesn't change with changes to the nominal dose rate of the linac. Therefore, changing the nominal dose rate cannot be used to verify or evaluate the dose‐rate dependence of a dosimeter or its suitability in the high‐dose‐rate environment of an FFF beam.

**Table 2:** Examples of measured $P_{\text{ion}}$ values at 300 V for Varian flattening filter‐free (FFF) beams at 10 cm depth in water and at $d_{\text{max}}$ with three different ion chambers.^(28)^

	*6 MV FFF*		*10 MV FFF*	
Chamber	10 cm	$d_{\text{max}}$	10 cm	$d_{\text{max}}$
Exradin A‐12	1.006	1.009	1.010	1.014
PTW TN30013	1.005	1.008	1.011	1.013
NEL 2571	1.008	1.013	1.015	1.018

Fourth, because the radiation profile is peaked on the central axis, large volume Farmer‐type ion chambers placed on the central axis will experience some partial volume averaging, and therefore underestimate the dose on the central axis. This effect was examined for Varian 6 and 10 MV FFF beams based on film‐measured profiles in Kry et al.^(28)^ Based on the dose falloff away from the central axis and the dimensions of Farmer‐type chambers, a partial volume effect was estimated to be 0.2% for both energies — that is, the ion chamber measurement would be 0.2% less than the true dose on central axis. This effect would be slightly larger if the ion chamber was not centered in the radiation field or if the beam was not properly steered. If the chamber was offset 5 mm from isocenter along the direction of the chamber (maximizing the size of the effect), the dose could be underestimated by up to 0.3% relative to the central‐axis measurements (for a 10 MV FFF beam).^(28)^ Partial volume effects and setup sensitivity would also be more pronounced at higher energies, at penumbra regions, or at regions of the profile with sharper dose falloff. Reasonable care should be used when centering the ion chamber. The AAPM Working Group on TG‐51 report recommends correction of the partial volume effect and/or the use of chambers with a short collecting volume to minimize partial volume effects.^(23)^

#### C.2 Profiles, depth doses, and other dosimetric properties

Commissioning and measurements of dosimetric properties should follow the general recommendations from TG 106,^(30)^ which contains detailed specification as to the scanning and non‐scanning data measurement requirements. A number of reports have descriptions of successful commissioning of FFF beams for various energies.^2^, ^3^, ^31^, ^32^, ^33^

For scanning measurements with an ion chamber, the variation in dose per pulse with depth and across the beam profile results in $P_{ion}$ varying with off‐axis position and depth (e.g., Table 2). The magnitude of the effect is dependent on variations in dose per pulse, as well as the chamber design (particularly the electrode spacing). While recombination, and therefore variations in recombination, will often be smaller for smaller scanning chambers, this may not always be the case. Changes in $P_{\text{ion}}$ would be expected to be less than 1% for many scanning chambers based on Table 2 data and other investigations.^(34)^ However, for a given model of scanning chamber, the range of ion recombination should be measured — between $d_{\text{max}}$ on the central axis (maximum dose per pulse) and at a relatively deep depth far off‐axis (minimum dose per pulse). Clinical judgment should then be used to determine if the variation in $P_{\text{ion}}$ is acceptable. If it is not acceptable, a different chamber or dosimeter should be used. For flattened beams, $P_{\text{ion}}$ also changes as a function of depth (or with wedges, for example). However, changes in $P_{\text{ion}}$ are not generally accounted for because $P_{\text{ion}}$ is very close to 1 and therefore variations in $P_{\text{ion}}$ are very small. For FFF beams $P_{\text{ion}}$ is larger, and therefore variations in $P_{\text{ion}}$ across the treatment field will be larger.

Dose‐rate effects may be relevant to other dosimeters, as well, and careful attention should be paid to any dose‐rate‐dependent device.

The commissioning scans of FFF beams will have different characteristics than are seen with flattened beams. The type and magnitude of these differences will depend on whether the energy of the electrons hitting the bremsstrahlung target has been increased (to restore the PDD), as with Elekta linacs, or not, as with Varian linacs. It should be noted that restoration of the PDD does not equate to equivalence of the underlying spectra and, while other properties may often be similar, they should not be expected to be identical. Below are presented major characteristics and the results that should be expected for FFF beams.

The PDD will be more shallow for FFF beams because the photon energy spectra are softer, unless the PDD has been restored through increasing the electron energy.^2^, ^35^ However, for all FFF beams, the PDD has minimal dependence on off‐axis position and, therefore, while the beam profile will show a bell‐shape, the shape of the profile will change only minimally with depth (off‐axis factors are minimally depth dependent).^2^, ^35^, ^36^ In flattened beams, profile shape varies with depth primarily because of spatially variant beam‐hardening, which does not occur for FFF beams.

Machine output measurements show less variation with field size for FFF beams than for conventional flattened beams. This was demonstrated by Vassiliev et al.^(2)^ who measured Sc,p for a range of field sizes. The decreased variability in output is attributed to decreased head scatter because the absence of the flattening filter removes a major scatter source.^(37)^ Naturally, phantom scatter factors are also different than for flattened beams.^(32)^

The penumbra for FFF beams has been found to be sharper than for flattened beams (particularly for small fields), although this is most clearly the case when the energy of the FFF beam was *not* raised (to restore the PDD).^32^, ^38^ When the energy was raised (as for Siemens and Elekta linacs), the FFF penumbra has been found to be comparable to, or broader than, the penumbra for flattened beams.^(32,32)^ In general however, these differences are small.

When the energy has not been restored, the softer photon spectrum results in less MLC leaf transmission.^32^, ^38^

For a Varian linac, skin dose for FFF beams has been found to be modestly higher compared to flattened beams, as measured with a parallel plate ionization chamber.^23^, ^39^ For example, the dose at 1 mm depth relative to $D_{\text{max}}$ was found to be 53% for a flattened 6 MV beam, but 61% for an FFF beam. Skin dose varies with field size; because there is less low‐energy contamination from head scatter, skin dose from FFF beams increases more slowly than for FF beams as field size increases. Consequently, skin doses from FFF beams may be comparable or even lower than for flattened beams for large fields.^3^, ^32^ Increasing the beam energy to restore the flattened PDD mitigates the increase in skin dose.^(32)^ In general however, further study of surface doses is warranted to more fully understand the impact of treatment parameters, the skin dose associated with clinical treatment conditions, and the reliability of the TPS in predicting skin dose in the plans for which FFF might be used. While several studies in the literature have estimated the skin dose, this has typically been at a depth of 3 mm, which generates substantially different doses than at the actual depth of the skin target cells (≤ 0.1 mm^40^, ^41^). Because skin dose measurements are difficult to make and treatment planning systems don't model this region accurately, we therefore recommend choosing beam arrangements that distribute the entrance dose over multiple ports, as is always good clinical practice.

Outside the treatment field, FFF beams generally produce lower dose.^1^, ^32^, ^42^, ^43^, ^44^ Leakage radiation and collimator scatter are substantially reduced. However, patient scatter is not decreased in the presence of a softer primary photon beam.^(42)^ Doses outside the treatment field are most reduced in FFF mode for smaller or highly modulated fields, such as stereotactic treatments, as head leakage is relatively important for these treatments.^1^, ^32^ When the energy of the electrons striking the target is increased (to restore the PDD as in the Elekta and Siemens implementations), the out‐of‐field dose is lower than when the electron energy is left unchanged.^(44)^

#### C.3 End‐to‐end testing

Task Group 142^(20)^ recommends end‐to‐end testing anytime new procedures are introduced. End‐to‐end testing helps ensure the safe operation of the entire system, including the treatment planning system, the R&V system, and the delivery system. For flattening filter‐free beams, areas of concern include accurate modeling of the flattening filter‐free beam by the TPS and proper transfer of the treatment plan to the delivery system, particularly the novel feature of the FFF beam designation. The current DICOM standard does not explicitly handle FFF beams and so vendors have adopted different schemes for encoding the FFF property. An end‐to‐end test, comprised of a CT scan of a phantom, transfer of the CT images to the TPS, generation of a clinical plan, transfer of the plan to the R&V system, and delivery of the plan, is therefore critical. The end‐to‐end test should follow the clinical process completely, handling the phantom in the same manner as a patient. This should also examine mixing conventional and FFF beams. At a minimum, point measurements should be made to verify the correct dose. An excellent resource for IMRT and VMAT end‐to‐end testing is the report of Task Group 119.^(45)^

### D. Periodic quality assurance

Development of a periodic quality assurance program is ultimately the responsibility of the site physicist, but must conform to applicable regulations, and should generally follow professional guidance documents, such as Task Group 142,^(20)^ although other established standards may also be acceptable.

Most QA procedures for FFF beams and linear accelerators are consistent with the QA that should be performed for traditional flattened beams. The vast majority of mechanical, safety, and even dosimetric properties of FFF beams and delivery systems are the same as for flattened beams, and the QA program can therefore largely follow traditional QA. Regarding the shape of the beam profile, TG‐142^(20)^ can be followed directly, establishing the “baseline” beam profile shape during commissioning. The profile shape can then be evaluated against this baseline on a point‐by‐point or area comparison basis (as is done for flattened beams), or by evaluating the slope of the profiles.^(46)^ However it is assessed, and similar to other FFF beams such as TomoTherapy,^(47)^ the consistency of the profile should be verified monthly (although more frequently may be easily achievable with some daily QA devices). The shape of the profile should also be compared to the treatment planning system data annually.

### E. Treatment planning

#### E.1 Planning systems

Modern treatment planning systems employ model‐based algorithms for dose calculation. Model‐based algorithms should be able to accommodate FFF beams; indeed the radially uniform FFF photon energy spectrum has been found to make modeling of such beams more accurate than the modeling of flattened beams.^(48)^ However, the specific software implementation must be designed to handle FFF beams, and the TPS vendor's instructions must specify the necessary beam data. Various commercial treatment planning systems are reported to have the capability to accurately handle FFF beams, including the Eclipse analytical anisotropic algorithm (Varian Medical Systems),^(49)^ Eclipse Acuros XB (Varian Medical Systems),^(49)^ Pinnacle collapsed cone convolution superposition (Phillips),^(31)^ CMS XiO (Elekta),^(31)^ Oncentra MasterPlan enhanced collapsed cone convolution (Elekta),^(48)^ Monaco Monte Carlo (Elekta),^(48)^ and Prowess Panther (Prowess Inc.).^(50)^ Other vendors should be consulted to determine the status of their support for FFF beams. The commissioning of FFF beams for treatment planning systems should follow standard guidelines.^45^, ^51^

#### E.2 Planning, optimization

For conventional treatment planning or forward treatment planning, the use of FFF beams may be more challenging than using conventional flattened beams because of the nonuniform intensity across the field. However, this difficulty is mitigated for small fields as intensity variations are minimal over the central 1‐2 centimeters of the treatment field.^(2)^ In general, neither patients nor tumors are flat; target coverage with FFF beams is readily achievable for small targets and may be achievable for larger targets, as well. Unmodulated FFF beams have been found to be suitable for small‐field procedures, such as SRS/SBRT, using 3D CRT.^1^, ^52^ FFF modalities should also be suitable for forward planned IMRT (e.g., field‐in‐field technique); however, as field size increases, forward planning may become more challenging. In general, FFF beams are not used for large, unmodulated fields.

Computer optimization, as used in inverse planning, removes the challenges of the nonuniform fluence. During IMRT planning, a typical broad beam is divided into many small beamlets to provide sufficient freedom of beam intensity variations required for IMRT. FFF beams have inherent intensity variations, but these are directly incorporated into the IMRT plan optimization. Several authors report that IMRT plan qualities are comparable when based on either flattened or FFF beams.^31^, ^53^, ^54^ FFF beams may sometimes produce slightly better target conformality and/or critical structure sparing,^(53)^ but mixed results have also been reported across different disease sites and with different energies.^(55)^ Consistently though, the differences have been found to be small and likely not clinically significant. Other treatment plan characteristics also change with the use of FFF beams. With FFF beams, it was found that more segments were required compared to IMRT plans with flattened beams for nonconcave tumor volumes and for large tumor volumes, although for small tumor volumes or highly concave tumor shapes, the number of segments in IMRT plans with FFF versus flattened beams were similar.^(54)^ Finally, FFF beams also require more monitor units to deliver treatments as, for off‐axis points, less than 1 cGy is delivered per MU.

It must be noted though that the specifics of 1 MU is different between flattened and FFF beams. One MU corresponds in both cases to the signal measured in the monitor chamber that corresponds to a dose of 1 cGy under reference conditions. However, for FFF beams, much less target current is required to produce the same signal in the monitor chamber. Correspondingly, less target current is required to deliver 1 MU, and less target current is required to deliver a course of treatment,^(56)^ even though more MUs are required.

## III. CLINICAL ISSUES

### A. Treatment plans and delivery

FFF beams have been implemented and evaluated for various disease sites. The main comparisons are based on delivery time and dosimetry. The increased dose rate allows for faster delivery, which may reduce the effects of patient and/or target motion, and increase patient comfort. The shortened delivery time will benefit some motion control techniques, such as breath‐hold. Treatments of small‐sized tumors benefit most as they have the most elevated dose rate. Dosimetrically, the quality of both stereotactic and IMRT treatment plans with FFF delivery is comparable to that using flattened beams. Dosimetric equivalence is not a surprising finding, given the long history of use and good performance of FFF beams in devices such as TomoTherapy.^57^, ^58^

Studies have also typically found that IMRT treatment planning time is independent of whether or not a flattening filter is in place.^59^, ^60^

### B. SRS/SBRT applications

The FFF modality was first investigated by O'Brien et al.^(1)^ for radiosurgery applications. More recently, and with a modern linac, Vassiliev et al.^(52)^ assessed the feasibility of stereotactic radiotherapy for early stage lung cancer using FFF photon beams from a Varian Clinac. Flattened beam 3D CRT treatments for 10 clinical patients were replanned with FFF beams. This study found that dose homogeneity in the target was not significantly different between treatments using flattened or FFF beams (p=0.3), and that doses to critical structures were comparable. The median beam‐on time per field was reduced from 25 s (with the filter) to 11 s (without the filter), increasing the feasibility of breath‐hold treatments and the efficiency of gated treatments. On a clinical machine, Gasic et al.^(55)^ evaluated 30 SRS/SBRT patients for FFF versus flattened VMAT. They similarly found negligible dosimetric differences, but a reduction in beam on time of 50%–75%. Prendergast et al.^(8)^ also highlighted the substantial time advantage of FFF beams for stereotactic treatments with a review of 27 CNS radiosurgery cases treated using FFF beams. Delivered doses ranged from 12 to 30 Gy, delivered in 1 to 5 fractions. Despite the large dose per fraction, the mean time the patient spent in the treatment room (from the beginning of imaging to the completion of treatment) was only 10:42 (min:s; range: 6:05–22:56), leading to the conclusion that single fraction and hypofractionated CNS SRS can be accomplished within a standard radiotherapy time slot.

On a more cautionary note, however, Ong et al.^(61)^ found that the faster delivery may accentuate interplay between breathing motion and leaf motion, which may be a concern in hypofractionated IMRT in the thorax and abdomen.

### C. IMRT applications

Numerous studies have evaluated the potential for FFF beams to deliver IMRT. Fu et al.^(54)^ first considered this, exploring treatment delivery time, and found that FFF plans require up to 50% less beam‐on time than corresponding flattened beam treatments based on IMRT of the head and neck and prostate. This reduction was not clinically significant with 2 Gy doses, although it could be for larger doses per fraction.^(54)^ Vassiliev et al.^(53)^ compared treatment plan quality for flattened beam versus FFF IMRT of the prostate at 6 and 18 MV with a Varian linac and the Eclipse treatment planning system. Similar work was also done by Stathakis et al.^(31)^ using Pinnacle's collapsed cone convolution superposition algorithm. Both groups found comparable plan quality regardless of the presence of the flattening filter, with no clinically significant difference in terms of organ at risk doses and target dose homogeneity.

Stathakis et al.^(31)^ also evaluated head and neck, brain, and lung treatments using flattened versus FFF 6 MV IMRT. Again, minimal differences were seen between the beams in terms of target coverage, target homogeneity, or organ at risk DVH values. Spruijt et al.^(62)^ evaluated breast therapy using FFF beams and also found comparable dosimetric results between flattened and FFF beams.

FFF beams can be used in conjunction with VMAT delivery techniques. Zwahlen et al.^(22)^ compared VMAT plans for the prostate using 6 MV and 10 MV flattened and FFF beams. All four beams delivered comparable dosimetric plan quality. Treatment time was reduced by 35% when a single arc was used, but there was no difference in treatment time when multiple arcs were used (as the linac always rotated at maximum speed). However, even for the single‐arc case, the reduction was only from 1.5 min to 1 min, reinforcing the findings of Fu et al.^(54)^ that the delivery efficiency advantage for FFF beams at conventional fraction sizes is small and not of clinical significance. Similar results were found by Subramaniam et al.^(63)^ who evaluated VMAT for the chest wall, by Nicolini et al.^(64)^ who evaluated VMAT for the esophagus, and by Gasic et al.^(55)^ who evaluated VMAT for the brain, prostate, and head and neck. All three studies found comparable dosimetric results between the flattened and FFF beams; Subramaniam et al.^(63)^ found some improved sparing of organs at risk with FFF beams, Nicolini et al.^(64)^ found a small increase in delivery efficiency with FFF beams, while Gasic et al.^(55)^ found reduced homogeneity in the target for FFF beams in the brain and head and neck.

### D. Treatment delivery verification

Verification of FFF planned doses has been conducted, typically in the form of IMRT QA. Stathakis et al.^(31)^ measured the dose distributions in an acrylic phantom using a PTW 0.125 cc ionization chamber and Kodak EDR2 film. The point dose measurements were within ±3% and planar film analysis showed reasonable agreement under qualitative evaluation. Salter et al.^(50)^ evaluated seven plans using the Delta^4^ device (ScandiDos) and found central axis dose agreement within 3%, and greater than 95% of pixels passing gamma (3%/3 mm). A multi‐institutional study analyzed QA results for 224 patients treated using FFF beams, using both static field IMRT and arc techniques, and found them within the specified limits of 3 mm distance to agreement and 3% dose difference. This study was notable in that four verification devices were used and only small variations were found among the devices.^(65)^ These findings are an important step for evaluating QA devices, as “inaccuracy of QA devices” was a substantial concern in the FMEA analysis (see Table 3 in section III.G). Before use, clinical physicists should be thorough in evaluating the dose‐rate characteristics of their QA devices, and analyze their passing behavior when using them for FFF beams. This can be done using a combination of literature review, vendor specifications, and direct measurement.

**Table 3:** Example FMEA analysis of beam delivery unique to FFF. Per TG‐100, a 1–10 scale is used, where Occurrence (O) ranges from 1 (almost impossible) to 10 (almost inevitable), Severity (S) ranges from 1 (minor annoyance) to 10 (lethal), and Detectability (D) ranges from 1 (highly detectable) through 10 (almost impossible to detect until it causes patient harm). The product of O, S, and D denotes the relative risk of that failure mode. Values presented in the table are suggestions only, pooled from authors on this report.

*Failure Mode*	*O*	*S*	*D*	*Risk Probability Number (product)*
Inaccurate calibration (e.g., error in $P_{ion}$)	2	5	6	60
Failure to account for excessive skin dose	5	6	4	120
Dose problems from low MU segments	3	4	4	48
Inaccuracy of QA devices	4	5	4	80
Wrong beam type selection due to confusing user interface in planning	3	4	4	48
Wrong beam type selection due to confusing user interface in delivery	2	6	3	36
Wrong beam type selection due to incorrect transfer from TPS and/or R V	2	6	2	24
Use of wedges or other devices for which FFF wasn't commissioned	2	6	4	48
Failure to catch problem during treatment due to fast delivery	3	5	5	75
Calibration error due to chamber placement off‐axis	2	5	6	60

### E. Limitations of FFF

The addition of FFF beams to modern linear accelerators requires additional commissioning and routine quality assurance. The Varian TrueBeam, for example, offers up to five clinical photon beams. The addition of FFF beams raises the question: “How many beams are needed to deliver high‐quality care while not overwhelming QA resources?” Could FFF beams completely replace conventional flattened beams, which would then prevent a large increase in the number of clinical photon beams maintained? There are some limitations to this idea:

1. To replace conventional flattened beams with FFF beams in situations where 3D conformal radiation therapy is appropriate generally requires intensity modulation of the FFF beam. For example, wedged or gently modulated fields are often used with flattened beams for whole breast therapy. While FFF beams can generate acceptable plans for the whole breast,^(62)^ beam modulation is generally required. At many institutions in the US, IMRT for breast would require a change in clinical practice with associated billing implications.
2. In situations where short planning time is important, such as palliative cases, flattened beam plans can be planned and delivered much faster than inverse plans.
3. There are many clinical situations, particularly in the treatment of deep tumors, where high‐energy beams are preferred to 6 or 10 MV beams. On most platforms, high‐energy FFF beams are not available.
4. Total body irradiation (TBI) treatments and other extended distance treatments, such as mantle fields and spinal fields for craniospinal fields, are generally made more complicated with FFF beams.

These issues could be addressed in a number of ways, such as including an internal optimization option to generate a flat beam profile, or the availability of high‐energy FFF beams. However, such options are not currently available, indicating value in maintaining at least one conventional flattened beam.

### F. Practical considerations

Implementation of FFF technology requires some special considerations, including: 1) dosimetric understanding of the technology; 2) the effective use of the technology; 3) requirement of resources to implement the technology; and 4) quality assurance and safety.

As part of commissioning, the clinical workflow of how FFF will be implemented should be carefully understood and documented for each step of treatment: immobilization, simulation, planning, localization, and delivery.

Since FFF technology is relative new, beam characteristics need to be carefully studied prior to clinical use. Suitable measurement devices are available, but may require new equipment purchases and extra resources and training of the staff to perform these tasks. Clinical application procedures and quality assurance procedures should be developed as early as possible. More importantly, the site should develop protocols about what kind of patient and treatment will fit the FFF technology. Since not every patient and every type of treatment will be suitable for FFF technology, each clinical site should develop protocols about the criteria for using FFF technology. For example, when it can be used for treatment techniques such as 3D CRT, IMRT, and VMAT, and when it can be used for treatment procedures such as SRS and SBRT.

### G. Safety considerations

There are unique safety considerations or “failure modes” that are particular to FFF, and therefore should receive additional attention to proactively examine special risks introduced by FFF, at least while this technology matures. While these failure modes and potential risks are not fully understood at this time, they include concerns with dosimetry and calibration, but also clinical workflow. Failure Modes and Effects Analysis (FMEA) has been suggested as a useful risk assessment method, with several FMEA evaluations published, and as recommended by TG‐100.^66^, ^67^, ^68^, ^69^, ^70^, ^71^, ^72^ The type and frequency of QA tests should depend on the likelihood of failure (O), the severity of the consequences if something goes wrong (S), and the detectability of the failure (D). The TG members have done the FMEA in Table 3 for new risks resulting from the introduction of FFF to an established clinical practice, but readers are encouraged to evaluate these and other risks based on their own practice and to re‐evaluate their risk estimates as they gain familiarity with FFF. These risks should also be put into the context of radiotherapy delivery with flattened beams. Ideally, risks associated with use of FFF beams would be part of a comprehensive departmental risk analysis, but an analysis of special potential risks of FFF treatments is an efficient way to get started.

An institution's radiotherapy program should be tailored based on such an FMEA analysis. For example, if skin dose is a concern, detailed measurements could be conducted or treatment planning policies could be enacted to ensure beams are broadly spread out over the patient. If QA device performance at high‐dose rates is uncertain, a detailed comparison of the QA device to a dose‐rate independent standard should be conducted. This analysis may also drive additions to an institution's periodic QA program. Of note, end‐to‐end testing is critical to increasing the detectability of many problems, particularly beam type selection errors and dosimetric problems, such as low MU segments.

### H. Facility planning

#### H.1 Workload estimates

Workload (total administered dose per week) could differ between flattened and FFF machines if the machine is used for a different application (particularly dedicated stereotactic use). However, for most clinical scenarios, patient throughput, and therefore workload, is unlikely to increase with the FFF mode because the bulk of treatment time is not beam‐on time. Naturally, this will be determined by clinical needs and distribution of machine resources.

#### H.2 Vault barriers

For Varian linacs, where the energy is not restored, primary barrier thickness is 10%–20% less for FFF beams, compared with flattened beams, because of the decreased transmission associated with its softer photon spectrum.^(73)^ Secondary barrier thicknesses are also 10%–20% less for FFF beams, compared with flattened beams. This is in part because of decreased and lower energy patient scattered radiation exiting the patient/phantom at most angles. Secondary barrier thickness is also less for FFF beams because of decreased head leakage levels. Only one‐half (6 MV) to one‐quarter (18 MV) as much head leakage occurs with FFF‐based treatments as compared to flattened beam treatments.^(73)^ This is particularly important for IMRT applications where head leakage plays a major role in vault shielding.

For machines with energy‐restored FFF beams, there is still a shielding benefit, although it is slightly less. Primary barrier thickness was found to be up to 8% less, while secondary barriers were found to be up to 11% less.^(74)^

Radiation survey results have confirmed lower doses associated with the FFF modality.^74^, ^75^

#### H.3 Neutrons

Neutrons are generally a concern only for energies above 10 MV; currently, only Siemens linear accelerators have such FFF beams.

The neutron spectrum is essentially constant, regardless of the presence of a flattening filter.^76^, ^77^ Consequently, neutron shielding requirements scale directly with fluence, which is substantially reduced with FFF beams.^76^, ^77^, ^78^ This is primarily due to the increased efficiency of photon delivery — fewer photons are generated to deliver the same dose to the patient, so correspondingly fewer photoneutrons are created. To a much smaller degree, reduction in the neutron fluence also occurs because the flattening filter is no longer a source of neutrons (the flattening filter produces ~ 10% of neutrons in a high‐energy (18−24 MV) Varian linac^76^, ^79^ and <1% in a 15 MV Siemens beam^(80)^).

Neutron shielding values of source strength (Q) and dose equivalent (H_0_) for FFF beams are nearly 80% lower (per photon Gy at $d_{\text{max}}$ for an 18 MV Varian linac).^(76)^ In cases with beam modulation, the neutron source strength and dose equivalent must be scaled by the modulation, which for clinical treatments corresponds to an approximately 70% reduction in neutron fluence and dose equivalent.^(78)^

In addition to the decreased shielding burden, the decreased neutron fluence also corresponds to a decreased personnel dose through room activation.^(75)^

In short, neutrons are at most a minimal issue, even for high‐energy FFF beams.

#### H.4 Recommendations: Facility planning and radiation safety

From a facility planning and radiation safety perspective, FFF beams are superior to flattened ones in that there is a smaller shielding burden for the FFF mode. However, current FFF beams are offered in conjunction with flattened beams, requiring shielding of those flattened beams, as well. Therefore, unless the FFF mode is intended to be predominantly used, shielding for the traditional flattened beams is likely optimal, offering prudent shielding for all machine applications.

## IV. THEORETICAL ISSUES

### A. Radiobiological aspects

The dose‐rate differences between treatments delivered by conventional beams (∼3–6 Gy/min) and by FFF (∼15–30 Gy/min) have prompted some new investigations,^81^, ^82^, ^83^, ^84^, ^85^, ^86^, ^87^ and a second look at experimental studies of earlier decades.^88^, ^89^, ^90^, ^91^, ^92^, ^93^, ^94^, ^95^, ^96^ The dose rates that are usually quoted for both conventional and FFF (see Table 1) are averages, as dose is delivered in ~ 2 μs wide pulses with instantaneous $d_{\text{max}}$ dose rates of approximately 1.7×102 Gy/s for conventional beams and approximately 7×102 Gy/s for FFF. According to theoretical estimates^(94)^ and early radiobiology experiments,^83^, ^96^, ^97^ FFF instantaneous dose rates are not expected to be high enough to affect cell survival through changes in the basic radiochemistry of radical recombination.

It has been suggested that shorter total treatment times may result in less sublethal damage repair and thus more efficient tumorcidal effects, but also more risk of normal tissue damage^(81)^ or conversely, that treatments of longer duration are less tumorcidally efficient.^82^, ^87^, ^88^, ^89^, ^90^, ^91^, ^92^, ^93^, ^94^ A few studies have evaluated cell survival *in vitro* in FFF beams, but with differing results.^98^, ^99^

Clinically, FFF beams are just one means of reducing fraction delivery time — and FFF on its own does not necessarily lead to the most drastic decreases in total delivery time. The anticipated treatment delivery time difference between FFF and flattened beams is often less than the difference between IMRT and conventional therapies, or between IMRT and modulated arc therapy (VMAT) therapies. Additional confounding effects that may accompany reduction of treatment time in clinical studies include such things as improved positioning accuracy and the frequent combination of FFF with sophisticated image guidance — both of which should tend to improve local control on their own. The basic radiobiology of increased dose rate is certainly a subject of academic interest, and interested readers may wish to consult the references of this section, as well as additional sources.^93^, ^100^, ^101^ But it is unlikely that the question of whether the radiobiology of the dose‐rate increase in FFF beams can change clinical outcomes can be unraveled from the ‘noise’ of the many other variables present in typical clinical outcomes studies.

## V. FUTURE NEEDS

Although extensive work has been done in many areas of study regarding FFF beams, research is still needed.

Dosimetry questions may arise, as many detectors (including ion chambers) are dose‐rate dependent and, therefore, may need different correction factors for FFF beams in order to achieve precision dosimetry.

For many of these issues, the impact of changing the primary beam energy requires attention. Most of the studies in the literature have used the same electron energy incident on the target for both flattened and FFF versions of the beam. The consequent softer photon spectrum produces, among other phenomena, a more shallow $d_{\text{max}}$, increased skin dose, and increased patient scatter. With the Siemens and Elekta implementation, the accelerating potential of the FFF beam is increased to restore the PDD. This may restore $d_{\text{max}}$ to a corresponding flattened beam level, but other factors such as skin dose and patient scatter require further study. Readers of the literature must pay particular attention to the electron accelerating potential of the study they are reading. Clearer delineation of photon beam properties as a function of electron energy is necessary for FFF studies.

Skin dosimetry, its dependence on treatment parameters, and skin doses in clinical treatments, requires further study. It has, to date, typically been assessed at a relatively deep depth, below where sensitive skin layers are generally assessed. The impact of treatment parameters also requires additional study.

The role of FFF beams in radiotherapy would also be more clearly defined with further study on intrafractional motion and its possible interplay with MLC and gantry motion.

Clinical issues also need additional evaluation. Although FFF beams have been shown to be suitable for essentially all IMRT situations evaluated, is there a subset of clinical cases for which FFF IMRT is optimal? Additionally, for conventional therapy, is there a simple way to deliver flat fields without developing an inverse planned solution?

Finally, radiobiological issues remain somewhat unresolved. Although different dose rates are unlikely to be relevant in terms of basic chemistry, cell repair mechanisms may be affected. This could impact both tumor control and normal tissue toxicity, although the clinical ramifications of this remain unclear, particularly in the context of 3D CRT versus IMRT versus VMAT, where treatment times also vary substantially.

Despite the unanswered questions, FFF technology has been adopted rapidly and recommendations are, therefore, provided in this document. Refinements to this technology and to clinical recommendations will no doubt occur as this technology matures.

## VI. RECOMMENDATIONS

Acceptance Testing (II.B)

1. Prior to measurements, evaluate dose rate dependence of measurement devices.
2. Follow guidelines of TG‐45 and TG‐142 except that, for flatness, measure beam profile for conformance to specs.

Commissioning (II.C)

1. Calibration: Follow standard calibration protocol (e.g., TG‐51) but account for beam differences (II.C.1):
  Determine $k_{Q}$ per protocol, but expect a slightly different value.
  Determine $P_{\text{ion}}$ with two‐voltage technique, but verify validity of this approach.
  Center the chamber, especially for higher energies.
  Correct for partial volume averaging or use a smaller chamber.
2. General beam data (II.C.2):
  Evaluate variation of scanning chamber $P_{\text{ion}}$ for off‐axis positions.
  Follow recommendations of TG106.
3. Conduct end‐to‐end testing of the system (II.C.3).

Periodic QA (II.D)

1. Follow guidelines of TG‐142, looking at profile consistency over time.
2. Test all QA measuring devices for dose‐rate characteristics.

Treatment Planning

1. Make sure your TPS is able to handle FFF beams (II.E).
2. In view of the uncertainties (both measurement and TPS calculation) regarding FFF skin doses, use beam arrangements that spread out the skin dose (II.C.2).
3. Before clinical use at a specific disease site, do comparative planning for several cases to assess the plan quality with flattened vs. FFF beams for the same case (B., C.).

Safety

1. Construct an FMEA table to evaluate the risk of failure modes and test modes that are of most concern (III.G).
2. Verify R&V support for FFF beams (II.C.3).

Facility planning (III.H)

1. Existing vaults for same nominal energy are adequate.
2. New vaults with mixed flattened and FFF use, either use traditional (flattened) shielding (conservative), or reduced shielding if FFF use is expected to be substantial.

## ACKNOWLEDGMENTS

We would like to thank Ms. Carey J. Myers for devoting a tremendous amount of her time in arranging all the references.
